# Supplementary material for: The Systems Biology Research Tool: evolvable open-source software
Source: BMC Syst Biol. 2008 Jun 29;2:55. doi: 10.1186/1752-0509-2-55 (PMC2446383; doi:10.1186/1752-0509-2-55)
Supplement: Additional file 1 — SBRT Archive. An archive of the current version of the Systems Biology Research Tool. [file 1752-0509-2-55-S1.zip › sbrt-1.4.0/doc/developers_guide/api/sbrt/shell/text/EqualsMapFormatV1.html]

EqualsMapFormatV1


|  |  |  |
| --- | --- | --- |
| **Overview** \| **Package** \| **Class** \| **Use** \| **Tree** \| **Deprecated** \| **Index** \| **Help** | |  |
| **PREV CLASS**   **NEXT CLASS** | **FRAMES**    **NO FRAMES**     **All Classes** |  |
| SUMMARY: NESTED \| FIELD \| CONSTR \| METHOD | DETAIL: FIELD \| CONSTR \| METHOD |  |


---


## sbrt.shell.text Class EqualsMapFormatV1<K,V>

```
java.lang.Object
  sbrt.shell.text.AbstractMapFormat<K,V>
      sbrt.shell.text.EqualsMapFormatV1<K,V>
```

**Type Parameters:**: `K` - the key type.: `V` - the value type.

**All Implemented Interfaces:**: Format, MapFormat<K,V>

---

``` public final class EqualsMapFormatV1<K,V> extends AbstractMapFormat<K,V> ```

This class is a concrete implementation of `MapFormat`.
The string representation of map entries is:

*key = value*.

Instances of this class are immutable.

**Author:**
:   This class was written and documented by
    Jeremiah Wright while in the Wagner lab.

---

**Constructor Summary**

`EqualsMapFormatV1(Formatter<K> keyFormatter,
Parser<K> keyParser,
Formatter<V> valueFormatter,
Parser<V> valueParser)`
  
          Constructs a new map format using the provided key and
value formats.

`EqualsMapFormatV1(SimpleFormat<K> keyFormat,
SimpleFormat<V> valueFormat)`
  
          Constructs a new map format using the provided key and
value formats.


| **Method Summary** | |
| --- | --- |
| `static EqualsMapFormatV1<java.lang.String,java.lang.String>` | `getBasic()`             Returns an `EqualsMapFormatV1` that does not format or parse the keys or values supplied to it. |
| `PipeListFormat<V>` | `getListFormat()`             Returns the format used for lists of values. |
| `EqualsMapEntryFormatV1<java.lang.String,java.lang.String>` | `getMapEntryFormat()`             Returns the format used for variable-value pairs. |
| `PipeSetFormat<K>` | `getSetFormat()`             Returns the format used for sets of variables. |

**Methods inherited from class sbrt.shell.text.AbstractMapFormat**

`formatKey, formatKeys, formatPair, formatPair, formatValue, formatValues, formatValues, getKeyFormatter, getKeyParser, getValueFormatter, getValueParser, parseAllKeys, parseAllPairs, parseAllValues, parseAllValues, parseKey, parsePair, parseValue`

**Methods inherited from class java.lang.Object**

`clone, equals, finalize, getClass, hashCode, notify, notifyAll, toString, wait, wait, wait`

**Constructor Detail**

### EqualsMapFormatV1

```
public EqualsMapFormatV1(Formatter<K> keyFormatter,
                         Parser<K> keyParser,
                         Formatter<V> valueFormatter,
                         Parser<V> valueParser)
```

:   Constructs a new map format using the provided key and
    value formats.

    **Parameters:**: `keyFormatter` - the formatter to use for keys.: `keyParser` - the parser to use for keys.: `valueFormatter` - the formatter to use for values.: `valueParser` - the parser to use for values. **Throws:**: `java.lang.NullPointerException` - if either argument is `null`.

---


### EqualsMapFormatV1

```
public EqualsMapFormatV1(SimpleFormat<K> keyFormat,
                         SimpleFormat<V> valueFormat)
```

:   Constructs a new map format using the provided key and
    value formats.

    **Parameters:**: `keyFormat` - the format to use for keys.: `valueFormat` - the format to use for values. **Throws:**: `java.lang.NullPointerException` - if either argument is `null`.


**Method Detail**

### getBasic

```
public static final EqualsMapFormatV1<java.lang.String,java.lang.String> getBasic()
```

:   Returns an `EqualsMapFormatV1` that does not
    format or parse the keys or values supplied to it. In other
    words, it returns an `EqualsMapFormatV1` with
    the most basic possible functionality. Empty strings are not
    allowed however.

    :   **Returns:**: an `EqualsMapFormatV1` that does not format or parse the keys and values supplied to it.

---


### getMapEntryFormat

```
public EqualsMapEntryFormatV1<java.lang.String,java.lang.String> getMapEntryFormat()
```

:   Returns the format used for variable-value pairs.

    :   **Specified by:**: `getMapEntryFormat` in class `AbstractMapFormat<K,V>`
    :   **Returns:**: the format used for variable-value pairs.

---


### getSetFormat

```
public PipeSetFormat<K> getSetFormat()
```

:   Returns the format used for sets of variables.

    :   **Specified by:**: `getSetFormat` in class `AbstractMapFormat<K,V>`
    :   **Returns:**: the format used for sets of variables.

---


### getListFormat

```
public PipeListFormat<V> getListFormat()
```

:   Returns the format used for lists of values.

    :   **Specified by:**: `getListFormat` in class `AbstractMapFormat<K,V>`
    :   **Returns:**: the format used for lists of values.


---


|  |  |  |
| --- | --- | --- |
| **Overview** \| **Package** \| **Class** \| **Use** \| **Tree** \| **Deprecated** \| **Index** \| **Help** | |  |
| **PREV CLASS**   **NEXT CLASS** | **FRAMES**    **NO FRAMES**     **All Classes** |  |
| SUMMARY: NESTED \| FIELD \| CONSTR \| METHOD | DETAIL: FIELD \| CONSTR \| METHOD |  |


---
